# Patient-centred innovation for multimorbidity care: a mixed-methods, randomised trial and qualitative study of the patients’ experience

**DOI:** 10.3399/bjgp21X714293

**Published:** 2021-03-23

**Authors:** Moira Stewart, Martin Fortin, Judith Belle Brown, Bridget L Ryan, Pauline Pariser, Jocelyn Charles, Thuy-Nga Pham, Pauline Boeckxstaens, Sonja M Reichert, GY Zou, Onil Bhattacharya, Alan Katz, Helena Piccinini-Vallis, Tara Sampalli, Sabrina T Wong, Merrick Zwarenstein

**Affiliations:** Department of Family Medicine;; Department of Family Medicine and Emergency Medicine, Université de Sherbrooke, Sherbrooke, Canada.; Department of Family Medicine;; Department of Family Medicine;; Department of Family and Community Medicine, University of Toronto, Toronto, Canada.; Department of Family and Community Medicine, University of Toronto, Toronto, Canada.; Department of Family and Community Medicine, University of Toronto, Toronto, Canada.; Department of Family Medicine and Primary Healthcare, Ghent University, Ghent, Belgium.; Department of Family Medicine;; Department of Epidemiology and Biostatistics, Western University, London, Canada.; Department of Family and Community Medicine, University of Toronto, Toronto, Canada.; Department of Community Health Sciences and Department of Family Medicine, University of Manitoba, Winnipeg, Canada.; Department of Family Medicine, Dalhousie University, Halifax, Canada.; Research and Innovation, Nova Scotia Health, Halifax, Canada.; School of Nursing and Centre for Health Services and Policy Research, University of British Columbia, Vancouver, Canada.; Department of Family Medicine;

**Keywords:** family practice, multimorbidity, mixed-methods research, patient-centred care, primary health care

## Abstract

**Background:**

Patient-centred interventions to help patients with multimorbidity have had mixed results.

**Aim:**

To assess the effectiveness of a provider-created, patient-centred, multi-provider case conference with follow-up, and understand under what circumstances it worked, and did not work.

**Design and setting:**

Mixed-methods design with a pragmatic randomised trial and qualitative study, involving nine urban primary care sites in Ontario, Canada.

**Method:**

Patients aged 18–80 years with ≥3 chronic conditions were referred to the Telemedicine IMPACT Plus intervention; a nurse and patient planned a multi-provider case conference during which a care plan could be created. The patients were randomised into an intervention or control group. Two subgroup analyses and a fidelity assessment were conducted, with the primary outcomes at 4 months being self-management and self-efficacy. Secondary outcomes were mental and physical health status, quality of life, and health behaviours. A thematic analysis explored the patients’ experiences of the intervention.

**Results:**

A total of 86 patients in the intervention group and 77 in the control group showed no differences, except that the intervention improved mental health status in the subgroup with an annual income of ≥C$50 000 (β-coefficient 11.003, *P* = 0.006). More providers and follow-up hours were associated with poorer outcomes. Five themes were identified in the qualitative study: valuing the team, patients feeling supported, receiving a follow-up plan, being offered new and helpful additions to their treatment regimen, and experiencing positive outcomes.

**Conclusion:**

Overall, the intervention showed improvements only for patients who had an annual income of ≥C$50 000, implying a need to address the costs of intervention components not covered by existing health policies. Findings suggest a need to optimise team composition by revising the number and type of providers according to patient preferences and to enhance the hours of nurse follow-up to better support the patient in carrying out the case conference’s recommendations.

## INTRODUCTION

Multimorbidity is common, even in younger ages than previously imagined, and especially for low-income groups.^1^^–^^3^ There is a growing international agreement for solutions to multimorbidity to exist in primary care settings and to be patient centred.^4^^,^^5^ However, patient-centred interventions for people with multimorbidity vary greatly, typically including both principles of partnership between the patient and health professional^6^^,^^7^ and aspects of system integration,^8^ as well as some technologies.^9^ Evidence of effectiveness is inconsistent,^8^^,^^10^^–^^12^ including that gleaned from six recent trials.^13^^–^^18^ It is becoming clear that work must be continued to improve the interventions;^19^ testing them and illuminating mechanisms of success and failure may assist with the making of such improvements^19^ so this study had two research goals:

- to assess the effectiveness of a flexible patient-centred innovation (which arose from real-world practice in the policy context of Ontario, Canada) in relation to relevant patient-reported outcomes; and
- to ascertain the contexts, and under what circumstances, the innovation worked or did not work for patients.

A concurrent triangulation mixed-methods study^20^ was mounted with two simultaneous components: a pragmatic^21^^,^^22^ trial for first aim and a qualitative study of patients’ experience for the second.

## METHOD

### Trial

#### Participant eligibility

Patients were eligible for the trial if they:

- were literate;
- were aged 18–80 years old;
- had never before received the intervention; and
- in the family physician’s (FP’s) clinical judgement, were cognitively intact and had ≥3 chronic conditions.^23^^,^^24^

The upper age limit of 80 years was chosen to minimise loss to follow-up because the participant had been admitted to an institution. The threshold of ≥3 chronic conditions was chosen for two reasons:

- this is likely to engender more burden for the patient and the FP than the other common definition of multimorbidity of ≥2 conditions; and
- as the patients had more medical needs, the researchers felt they had the potential for a greater level of improvement in outcomes.

The number of chronic conditions was validated with the patient’s self-report in the baseline questionnaire.

**Table:** How this fits in

Patient-centred interventions for patients with multimorbidity have shown mixed results to date, so there is a need to help improve them. The present study indicated that the intervention implemented had a neutral impact on primary outcomes. Given the subgroup results in which the intervention showed improvements only for patients with an annual income of ≥C$50 000, the qualitative findings, and the fidelity assessment, policymakers and clinicians are encouraged to seek ways to enhance care for patients with annual incomes of <C$50 000, to optimise team composition based on an individual patient’s preferences and abilities, and to enhance and tailor follow-up care by ensuring the creation of a coherent plan with actionable steps.

#### Design and setting

A pragmatic randomised trial was conducted with nine team-based family practices that were familiar with the intervention (along with solo practices and emergency departments affiliated with those teams) in Toronto, Ontario, Canada. The provincial policy context emphasised innovations for complex patients with high healthcare utilisation.^25^

Ethics approval was received and the protocol has been published.^26^ The trial was registered in Ontario, Canada (NCTO2742597), and the CONSORT guidelines for pragmatic trials were followed for reporting methods and results.^27^

#### Intervention development

A patient-centred, multi-provider case conference — the Telemedicine IMPACT Plus (TIP) programme — was developed by the providers, then chosen by the researchers from six programmes identified in a province-wide environmental scan. The intervention was chosen because of its lengthy development and preliminary evaluation. It was developed in a way that aligned with guidelines for complex interventions,^28^^–^^30^ covering theory, gaps, pre-trial evaluations, and adaptation over time, and contained two key theoretical underpinnings — namely a patient-centred process^6^ in the patient–provider interaction and an integrated version of the Chronic Care Model.^31^

Two evidence gaps from a scoping review^32^ that had been conducted by the team were lack of empowering patient-centred communication and lack of integrated care among multiple providers in the context of multimorbidity care. Pre-trial evaluations of early versions, which had a longer face-to-face component but less follow-up support, of the intervention revealed enablers of and barriers to patient and provider enthusiasm relating to:

- time and scheduling of the multiple providers;^33^
- patient need for support to implement recommendations;^33^
- patient-centred agenda setting;^34^
- the process of identifying and inviting patients;^34^ and
- remuneration of providers.^34^

Over a period of 10 years, the intervention was shortened, provided a telemedicine (video) option in addition to the face-to-face option, and remuneration was negotiated.

The patient-centred invitation to patients to engage in the discussion, which was not explicit before, was made explicit and was honed: ‘What are your goals for this session?’ was asked in the context of improved agenda setting.

#### The TIP programme

A nurse, hired by the programme, met with each patient face to face for approximately 1 hour to understand what mattered to them, and then planned and coordinated a case conference of approximately six providers relevant to that patient from the following:

- FP (known to the patient);
- internist;
- psychiatrist;
- social worker;
- physiotherapist;
- occupational therapist;
- pharmacist;
- dietitian; and
- home care case manager.

In preparation for the case conference, the nurse accessed the patient’s file from the FP and forwarded all relevant medical and social history to the intervention team. The providers met (face to face or by video) with the patient for 1–1.5 hours so all parties could, through mutual collaboration, focus on the patient’s goals and develop an agreed care plan. Follow-up was provided by the nurse to help execute the recommendations over the subsequent 4 months. A full description is shown in Supplementary Box S1 using the TIDieR Checklist.^35^

#### TIP and the literature

The literature describes three main intervention types: patient-oriented, organisational, and training interventions.^8^^.^^36^ Combining 47 trials from two reviews,^8^^,^^36^ the researchers found that patient-oriented interventions were tested in 36% of trials, organisational interventions were tested in 51% of trials (of which all also had a patient-oriented component), and training interventions were tested in 13% of trials; training was not an element of the intervention presented here. The focus on patient goals was a common thread in all the patient-oriented interventions; however, TIP’s multi-provider team was unique and not found in any of the 47 interventions — the closest thing being a team comprising the practice nurse, a psychologist, and a psychiatrist.^37^

#### Pilot evaluation of the TIP programme

A pragmatic randomised pilot study to determine the feasibility of suggested outcomes, to estimate recruitment, and to identify effect sizes for sample size calculation was conducted.^38^

#### Description of usual care

Patients in the control group received usual care in the office of their FP (typically a 15-minute visit) and a one-page list of community resources that patients with their conditions could contact if desired. The three-quarters of patients were referred by FPs who worked in an interprofessional team on site and a quarter were not. The three-quarters worked in Ontario’s model of team-based care, called the Family Health Team (FHT), which meant that a nurse practitioner and social worker would be readily available on site; the patients comprising a quarter of the control group were referred by FPs who were in a non-team practice but who could refer patients to medical specialists and health professionals off site.

#### Recruitment

Nine team-based practices and their affiliated practitioners referred eligible patients. There was a two-step recruitment process. Patient selection was based on clinical judgement and clinicians approached patients and requested consent to send their name to the researchers. The project coordinator received names and contact details of patients, and contacted them by telephone to explain the project in detail and obtain signed consent to participate.

#### Outcomes

Outcomes were assessed at baseline and at 4 months after the case conference, a period considered long enough for the nurse and patient to complete the plan and feasible for follow-up to the trial. Two primary outcome measures were chosen to represent patient education, empowerment, and agency:

- the Health Education Impact Questionnaire (heiQ);^39^ and
- the Self-Efficacy for Managing Chronic Disease scale (SEM).^40^

There were four secondary outcome measures:

- VR12 Health Status — Physical Component Score and Mental Component Score;^41^
- EQ-5D quality of life;^42^
- Kessler Psychological Distress Scale;^43^ and
- Health Behaviour Survey.^44^

Psychometric properties are available in the protocol article for the project.^26^

#### Sample size

For the two primary outcome measures (heiQ and SEM), comparing mean scores to detect a medium effect size (0.5) with a two-sided α = 0.05 and 80% power resulted in 64 participants being needed in both the intervention group and the control group; this equated to a sample size of = 128.^45^ Allowing for a 15% drop-out, the researchers aimed to recruit 150 patients, with 75 in each group.

#### Randomisation

Individual patients were allocated using randomly sequenced envelopes. Supplementary Box S2 details the procedures using the CONSORT guidelines^27^ regarding assignment of the intervention, sequence generation, allocation concealment, implementation, blinding, and data collection.

#### Statistical methods

Outcome data were analysed using the mixed model for repeated measures (MMRM).^46^ This method is a simple form of mixed effect; it does not explicitly model the random effects but, rather, explicitly models correlations among measurements within a subject. An advantage is that it can effectively handle missing data without strong assumptions that missing data occurred randomly. It also controlled for the baseline outcome measure. Nonetheless, a sensitivity analysis, omitting the lost cases, was conducted.

Two exploratory, post-hoc, subgroup analyses were conducted, on:

- <$50K annual income versus ≥$50K annual income (the sample’s median income); and
- ≥6 morbidities versus fewer (again, using MMRM).

In addition, the relationship between the fidelity of the intervention (involving a subset of 40 patients from the intervention group) and outcomes was analysed using analysis of covariance, controlling for baseline.

### Qualitative study

#### Design

A thematic analysis^47^ was used to explore the patients’ experiences of context, process, and under what circumstances the intervention worked or failed to work. It was undertaken at the same time as the trial measures were being collected.

#### Participant recruitment and final sample

Participants were selected purposively from those in the trial intervention arm. A maximum-variation sample varied by age, sex, and practice.

#### Data collection

Participants were interviewed 4 months after their TIP case conference at a time and date organised by the study research coordinator. Interviewers had no prior relationship with participants. Before the interview, participants read the letter of information that outlined the reasons for the qualitative study and informed consent for the interview was obtained.

Semi-structured individual interviews (Supplementary Box S3) were conducted with each participant alone in their home or the FP’s office. These lasted 30–60 minutes, and were audio-recorded and transcribed verbatim. Four members of the research team trained in conducting qualitative interviews undertook the collection and analysis of the qualitative data; they were not involved in the collection of the trial data.

#### Data analysis

The data analysis was both iterative and interpretive. All transcripts were independently reviewed and coded by the four researchers to determine the key emerging concepts. They then met, shared, and created a consensus that informed the development of the coding template. This process continued until no new themes were identified; the data were input into NVivo 10. In the final step, the research team identified overarching themes and exemplar quotations for each theme.

Trustworthiness and credibility were ensured using audio-recordings, verbatim transcripts, independent as well as team analysis, and field notes following each interview. A commitment to reflexivity considered how the researchers’ professional backgrounds (for example, social work, epidemiology, family medicine), particularly during the coding and interpretation of the data, could influence the findings.

## RESULTS

### Trial

#### Participants

**Figure 1.**
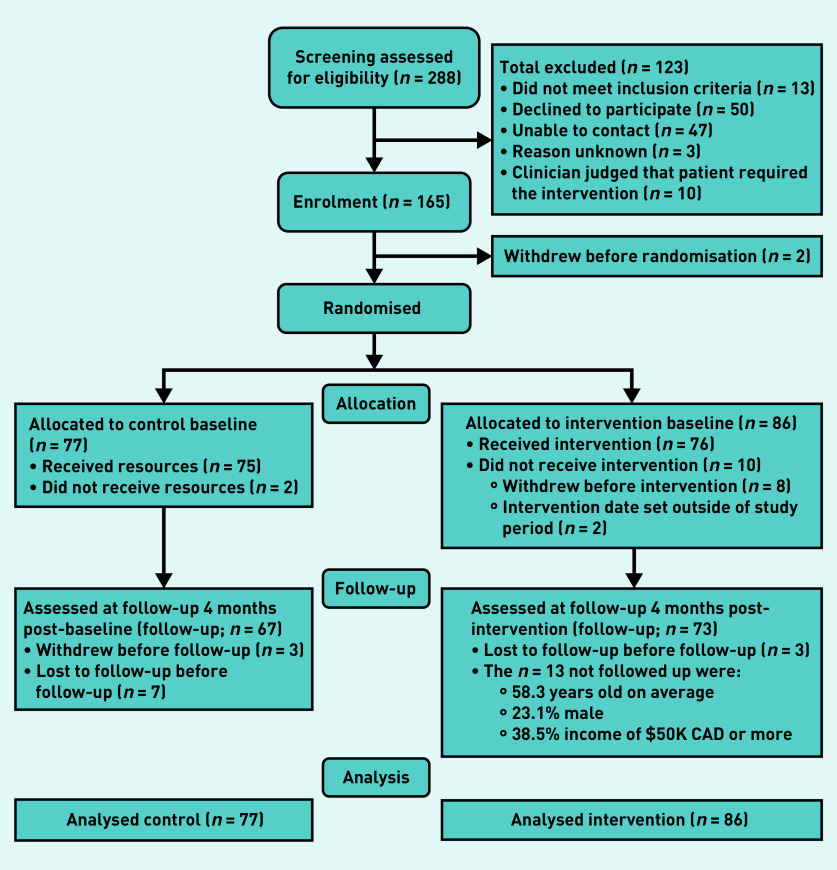
**Flow chart of patients.**

Figure 1 shows the flow diagram of the patient recruitment process. The sample attained was 86 for the intervention group and 77 for the control group. This sample differed from the general Ontario population^48^ in that it was older, comprised more females and participants had a higher level of education and a higher income.

Baseline patient characteristics are shown in Table 1. As expected, due to randomisation there were no statistically significant differences.

**Table 1.** Patient baseline characteristics of the intervention (*n* = 86) and control (*n* = 77) groups

	**Intervention**	**Control**	***P-value***
**Age in years, mean (SD)**	61.9 (13.9)	63.1 (13.9)	0.941

**Chronic conditions,^a^**			
No. conditions per participant, mean (SD)	6.1 (2.5)	5.9 (2.3)	0.414
**Type of condition, *n* (%)**			
Arthritis or rheumatoid arthritis	51 (59.3)	45 (58.4)	0.936
Depression or anxiety	49 (57.0)	40 (51.9)	0.497
Hypertension	47 (54.7)	40 (51.9)	0.253
Chronic musculoskeletal	46 (53.5)	29 (37.7)	0.027
Stomach problems	42 (48.8)	36 (46.8)	0.518
Colon problems	35 (40.7)	24 (31.2)	0.167
Hyperlipidaemia	33 (38.4)	29 (37.7)	0.412
Asthma or COPD	32 (37.2)	29 (37.7)	0.644
Cardiovascular disease	31 (36.0)	35 (45.5)	0.595
Diabetes	30 (34.9)	28 (36.4)	0.861
Thyroid disorder	22 (25.6)	17 (22.1)	0.566
Osteoporosis	20 (23.3)	20 (26.0)	0.773
Chronic urinary problem	13 (15.1)	13 (16.9)	0.955
Stroke or TIA	13 (15.1)	9 (11.7)	0.390
Heart failure (valve problem or replacement)	12 (14.0)	12 (15.6)	0.913
Cancer in previous 5 years	10 (11.6)	12 (15.6)	0.668
Kidney disease or failure	9 (10.5)	7 (9.1)	0.616

**Male, *n* (%)**	29 (33.7)	27 (35.1)	0.632

**Education level, *n* (%)**			
Incomplete secondary school	10 (11.6)	8 (10.4)	
Completed secondary school	9 (19.5)	11 (14.3)	
Some university or completed college	26 (30.2)	27 (35.1)	0.755
University (undergraduate or above completed)	40 (46.5)	31 (40.3)	

**Household income, *n* (%)**			
<C$20 000	20 (23.3)	17 (22.1)	
C$20 000–49 999	13 (15.1)	22 (28.6)	0.203
≥C$50 000	42 (48.8)	29 (37.7)	
Missing data	11 (12.8)	9 (11.7)	

**Marital status, *n* (%)**			
Married	37 (43.0)	37 (48.1)	
Separated or divorced	17 (19.8)	15 (19.5)	0.494
Widower	8 (9.3)	11 (14.3)	
Never married	23 (26.7)	14 (18.2)	

**Employment, *n* (%)**			
Employed	17 (19.8)	14 (18.2)	
Unemployed	31 (36.0)	28 (36.4)	0.967
Retired	37 (43.0)	34 (44.2)	

a HIV and chronic hepatitis not included as cell counts were < 5, in spite of their inclusion in the list of chronic conditions.^22^ COPD = chronic obstructive pulmonary disease. SD = standard deviation. TIA = transient ischaemic attack.

#### Outcomes and estimates

Table 2 shows the intent-to-treat analyses of primary outcomes in the intervention and control groups at 4 months; no statistically significant differences are in evidence. Tables 3 and 4 shows similar lack of difference on the secondary outcomes. Tables 2–4 show for the most part modest improvements from baseline to 4-month follow-up in both groups.

**Table 2.** Intention-to-treat analyses of primary outcomes at 4-month follow-up in the Telemedicine IMPACT Plus study

	**Intervention^a^**		**Control**			
	
	**Baseline**	**Follow-up**	**Baseline**	**Follow-up**	**β-coefficient (95% CI)^b^**	***P*-value**
Participants, *n*	86	73	77	67		

heiQ outcomes,^c^ mean (SD)						
Health-directed behaviour	2.72 (0.748)	2.86 (0.586)	2.83 (0.725)	2.93 (0.579)	0.049 (−0.149 to 0.247)	0.626
Positive, active, engaged life	2.90 (0.617)	2.93 (0.531)	2.84 (0.587)	2.95 (0.506)	−0.067 (−0.231 to 0.098)	0.429
Emotional wellbeing	2.42 (0.744)	2.56 (0.581)	2.45 (0.712)	2.61 (0.592)	−0.018 (−0.189 to 0.153)	0.836
Self-monitoring and insight	3.07 (0.372)	3.10 (0.277)	3.13 (0.355)	3.14 (0.305)	0.024 (−0.090 to 0.138)	0.681
Constructive attitudes and approaches	2.90 (0.644)	2.88 (0.464)	2.87 (0.572)	2.96 (0.526)	−0.108 (−0.264 to 0.048)	0.174
Skill and technique acquisition	2.75 (0.512)	2.83 (0.366)	2.76 (0.430)	2.94 (0.420)	−0.082 (−0.219 to 0.055)	0.243
Social integration and support	2.80 (0.679)	2.85 (0.499)	2.68 (0.647)	2.87 (0.524)	−0.152 (−0.309 to 0.005)	0.057
Health services navigation	3.13 (0.482)	3.11 (0.408)	3.11 (0.475)	3.17 (0.455)	−0.064 (−0.207 to 0.079)	0.381

Self-Efficacy for Managing Chronic Disease scale score^d^	5.69 (2.269)	5.93 (2.057)	5.59 (2.199)	6.06 (2.114)	−0.184 (−0.831 to 0.463)	0.577

a A sensitivity analysis, omitting the 10 patients who did not receive the intervention, was conducted and obtained similar results.

b Analysis by mixed-model repeated measures using Stata, version 13.

c Range: 1 (low)–4 (high).

d Range: 1 (low)–10 (high). CI = confidence interval. heiQ = Health Education Impact Questionnaire. SD = standard deviation.

**Table 3.** Analysis of secondary continuous outcomes^a^ at 4-month follow-up

	**Intervention**		**Control**			
	**Baseline**	**Follow-up**	**Baseline**	**Follow-up**	**β-coefficient (95% CI)^a^**	***P*-value**
Participants, *n*	86	73	77	67		
Health status, mean (SD)^b^						
Physical	34.09 (11.851)	36.61 (10.670)	34.16 (10.474)	37.05 (11.995)	0.274 (−2.775 to 3.323)	0.860
Mental	42.66 (13.592)	43.86 (13.506)	44.01 (13.783)	46.23 (13.119)	−1.402 (−5.055 to 2.251)	0.452
Quality of life	0.66 (0.247)	0.66 (0.257)	0.67 (0.246)	0.64 (0.237)	0.037 (−0.034 to 0.109)	0.307

a Analysis by mixed-model repeated measures, using Stata (version 13).

b Range: 1 (low) to ≥66 (high). CI = confidence interval.

**Table 4.** Analysis of secondary dichotomous outcomes^a^ at 4-month follow-up

	**Intervention**		**Control**			
	
	**Baseline**	**Follow-up**	**Baseline**	**Follow-up**	**Odds ratio (95% CI)^a^**	***P*-value**
Participants, *n*	86	73	77	67		

Psychological distress, %	18.60	15.49	19.48	13.64	1.490 (0.241 to 9.197)	0.668

Health behaviours^b^						
No alcohol	42.86	39.73	42.86	38.81	1.161 (0.143 to 9.453)	0.889
Physical activity 2 times per week	41.86	57.53	53.25	59.70	2.197 (0.595 to 8.110)	0.237
Good–excellent healthy eating	52.94	63.01	61.04	67.16	1.792 (0.362 to 8.882)	0.475
Healthy BMI^c^	62.65	56.94	60.81	56.92	0.475 (0.066 to 3.432)	0.461

a Multi-level mixed-effects logistic regression using Stata (version 13).

b Yes = healthy behaviour.

c <30. BMI = body mass index. CI = confidence interval.

#### Ancillary analyses

**Figure 2.**
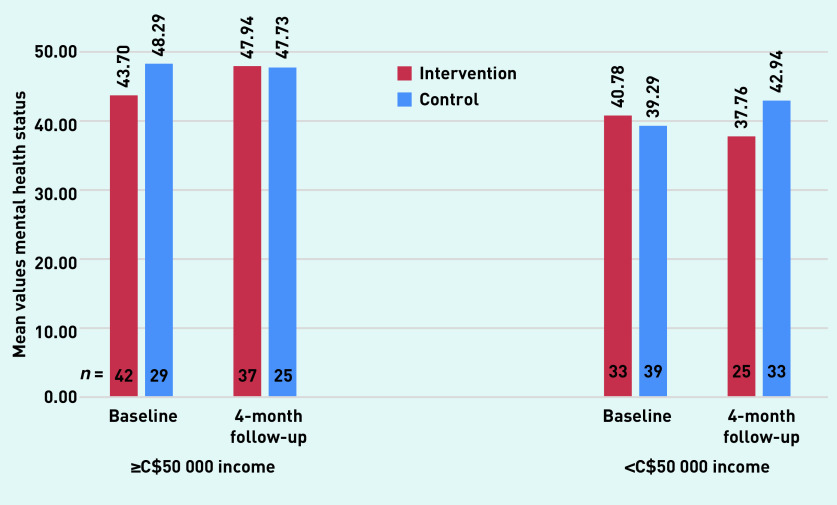
***Intervention and control group in relation to mental health status****^a^* ***by income subgroups.****^a^****Mean values of mental health status in a significant three-way interaction between treatment by time by income in an analysis by mixed model for repeated measures using Stata v. 13 (β-coefficient = 11.003,* P *= 0.006).***

Subgroup analyses showed no difference in the effect of the intervention for patients with ≥6 chronic conditions versus <6 (data not shown); however, as shown in Figure 2, the intervention effect differed significantly in the two income groups. The intervention group’s mental health status increased in the ≥C$50 000 patients and the usual-care control group’s increased in the <C$50 000 patients (β-coefficient 11.003, *P* = 0.006). Exploratory analyses regarding the fidelity of the interaction revealed two components that could be altered to improve the intervention, as follows (data not shown):

- having ≥3 hours (versus fewer hours) of nurse follow-up work within 4 months of the case conference was related to statistically significantly less improvement in primary outcomes from baseline to 4-month follow-up; and
- having ≥6 healthcare providers involved in the intervention (versus <6 providers) was related to less improvement.

### Qualitative study

The final sample comprised 14 patients (six men, eight women), aged 33–80 years. Five themes reflected the patients’ experience of the intervention:

- valuing the team experience;
- feeling supported in meeting their goals;
- receiving advice and a follow-up plan;
- being offered new and helpful additions to their treatment regimen; and
- experiencing positive outcomes.

#### Valuing the team experience

This theme included all players sharing the same information and having buy-in to the recommended plans, which participants felt was valuable:

‘ *The nurse got together a dietitian, my family doctor, a social worker, a psychiatrist, a pharmacist — a whole bunch of people together … We had a video here* [at my home] *, a conversation and just so everybody was on the same page with what I was doing. It was really good.’*

(Participant 4, female)

In addition, the diversity of team members meant connections to a lot of programmes could be made, which was found to be helpful:

‘Because I have various health issues, this gave me some kind of all-in-one resource!!’

(Participant 11, male)

However, a down side was that the new services could be exhausting:

‘ *I had so many appointments after* [the consultation] *, it was tiring!!’*

(Participant 4, female)

#### Feeling supported in meeting goals

The multi-provider case conference made patients feel that they were supported in terms of meeting their goals:

‘I felt that they truly were committed to the interview and looking to see if they could help me to reach my goals … It was often they’d have a perspective and then I’d respond to that as well. Or they’d ask me how I felt about that.’

(Participant 8, female)

In addition, their goals were explicitly elicited:

‘I was asked, “What do you want from this?” So I said, “I would like to be able to walk a mile … I am not asking to run a marathon, just a mile without pain”.’

(Participant 7, male)

Patients felt validated during the case conference:

*‘Well they heard me … they validated me. So that wasn’t happening* [before] *.’*

(Participant 4, female)

#### Receiving advice and a follow-up plan

The advice given was considered plentiful, new, and helpful:

*‘I felt really good coming out of that. That was some of the best answers* [ *sic*] *I felt like I’d received up to that point … I came out of it with kind of a list of ideas of different things to try to help improve my condition.’*

(Participant 1, male)

In some instances, patients felt it was the best part of the programme.

Patients appreciated receiving a summary of the recommendations from the consultation:

*‘The nurse came back* [to the patient’s home] *a week after that with a synopsis of everything that was written down and reviewed everything, not word for word, but the highlights, a summary of everything.’*

(Participant 7, male)

A few patients noted that, when presented with only verbal instructions or a list that was not coalesced into a plan, they were left with little guidance at the conclusion of the consultation:

‘It has been frustrating not to have a little bit more guidance about how to take the individual suggestions and put those into an actual plan … The list I was given seemed to be in the order they thought of them and not really further processed into an actual treatment plan with step-by-step priorities.’

(Participant 12, female)

#### Being offered new and helpful additions to their treatment regimen

Patients described how receiving new solutions to their health issues enhanced their treatments in spite of some treatments not being covered by the provincial healthcare plan:

‘Clear solutions yeah … a walker … medication … eating … that pleases me very much …’

(Participant 12, female)

‘As a result of this intervention, the social worker arranged for me to get a walker. So I go out for walks now, which I hadn’t been doing for the longest time. I find it very liberating.’

(Participant 5, male)

Psychotherapy and physiotherapy were repeatedly mentioned as helpful additions to patients’ treatment regimens:

‘The psychotherapist is a good match for me and positive work is being done. I am over the moon, optimistic.’

(Participant 13, female)

‘I feel like the physiotherapist has been helping. Yeah, anything that gets your strength back is wonderful … Especially muscles, because you can get them back, and I didn’t know how to do it.’

(Participant 12, female)

#### Experiencing positive outcomes

Participants described their health improvements, attributed to the programme, which reflected two dimensions — namely, improved functional ability and a new positive, hopeful attitude:

‘So I have had some improvement in being able to do things around the house.’

(Participant 12, female)

For some patients, increased function was due to a decrease in pain:

‘I’m feeling better. I don’t have the horrible pain that I had before … I feel that I have the ability to do things now that a few months ago I wouldn’t think of doing.’

(Participant 5, male)

Improved functional ability appeared linked to a sense of hope:

‘There are things that I’m looking forward to, which before the intervention I wasn’t.’

(Participant 5, male)

‘Without it, I would be sitting here miserable … there would have been no hope.’

(Participant 3, female)

## DISCUSSION

### Summary

The trial found the intervention had no statistically significant effects on the primary outcomes, although one subgroup (those with an income of ≥C$50 000) significantly benefitted in terms of the mental health outcome. Qualitative and fidelity findings revealed aspects of the intervention that could be improved.

### Strengths and limitations

One strength was that the intervention was developed by providers and pre-tested before the trial. Intervention failure was not a problem here as it has been with other interventions.^19^ Co-creation of the intervention, as in Mercer *et al*,^15^ or provider creation, as in the present study, appear to avoid implementation lapses.

A second strength was that the intervention includes an explicit patient-centred component, whereas most intervention studies to date, addressed the organisation of care.^8^^,^^32^

A third strength was the mixed-method design providing insights into how and for whom it works or fails to work; this is important given the neutral or mixed results revealed in recent systematic reviews^8^^,^^10^^–^^12^ and six recent trials.^13^^–^^18^

Other strengths include susceptibility bias, which was avoided by using envelopes in random sequence, while detection and follow-up bias was avoided by using sealed and opaque envelopes, and having a different staff member administer patient questionnaires.

One limitation was the fact that the sample size was relatively small in comparison with other recent trials (although the estimated sample size of 128 was met) and that the sample was unrepresentative of the general population in Ontario, Canada.

A second limitation was the lack of cost analyses. Healthcare utilisation outcomes, with their associated cost implications, will be handled in separate analyses that are underway.

Third, the qualitative study was not a full process evaluation. Therefore, future studies may want to include a process evaluation involving patients plus providers and decision makers.

Fourth, lack of alignment of outcome measures with intervention goals and patient expectations may be a problem.

Fifth, fidelity variations in some components of the intervention may have compromised impact.

### Comparison with existing literature

The current study advances on previous work by testing an intervention that should enhance everyday practice. This intervention should overcome the problem of implementation lapses found in other studies,^19^ because it was provider created, which goes even further than a co-created intervention.^15^ The lack of impact on outcomes for patients with an income of <C$50 000 contrasts with Mercer *et al* ’s^15^ multifaceted intervention, which was effective for patients who were considered to be socioeconomically deprived; they focused on self-management support, more face-to-face time with patients, and continuity of care — aspects of care that could enhance follow-up to the TIP programme. A systematic review^8^ found improvements in mental health outcomes, as did the present study.

### Implications for research and practice

Three possible explanations are offered for the neutral results in the pragmatic trial, each of which has implications for practice and research: the usual care received by the control group; the outcome measures; and the intervention itself. Each will be considered in turn. The first possible explanation is that usual care received by the control group may have been beneficial. The authors noted that the majority of control group patients experienced usual care within an Ontario-wide enhanced primary care team model, with interdisciplinary providers and continuity of care. The implication for practice is that some patients may benefit from the features of usual care whereas others may benefit from the multi-provider case conference, and future research can assist family physicians in their selection of which care is beneficial for which patients.

With regard to outcome measures, the trial presented here found a positive impact on the mental health outcome for a subgroup of patients and the qualitative findings focused on patients’ functional goals, as well as patients feeling validated, liberated, optimistic, and hopeful. Future research could use existing measures or develop new measures of mental health, patients’ function, and patients feeling validated.

With regard to the intervention itself, the qualitative findings and the fidelity assessment point out two aspects of the intervention that could be strengthened to enhance patient outcomes. Having ≥6 providers in the case conference was linked to negative outcomes, suggesting that the ideal/optimal team composition may need to be tailored to patient preferences — tailoring has been proposed by others.^18^ The number of nurse hours during the followup period, and how these hours are used, may need rethinking; such a rethink could involve increasing the involvement of the FP and the multidisciplinary team in the follow-up to better support the patients. Both of these findings regarding the number of providers and nursing hours imply a level of complexity that led to patient frustration (qualitatively); solutions to that may be specifically coalescing the plan into written, actionable steps and more support to the patient in carrying out the plan.

Patients qualitatively experienced promising effects of the very recommendations that required an extra outlay of costs — that is, those not covered by Ontario, Canada’s universal healthcare system — such as physiotherapy and psychotherapy. As well, patients’ income was implicated in the trial. In Toronto, 36% of people have an income of ≥C$50 000 and 64% <C$50 000.^49^ The ≥C$50 000 income group seemed to benefit from the intervention and those <C$50 000 seemed to benefit from usual care. The result, if replicated, could influence decisions in practice such as advocating to ease costs for a lower-income patient, and should encourage policies to cover such services.

In practice, the intervention could be improved by optimising team composition to align with individual patient preferences and reducing any frustrations for patients by having a clear care plan with actionable steps. For the subgroup of patients experiencing deprivation, additional supports such as identifying sources of financial assistance will be needed.

In research, the differences between income groups need to be replicated. Also piloted, provider-created interventions could be trialled to diminish the difficulties of implementation experienced by some researchers.^19^

The study findings support a suggestion that future research assessing interventions for multimorbidity include outcome measures on mental health, patients’ function, and patients feeling validated.

In policy, equity in patient-centred multimorbidity care may become an increasingly compelling issue.
